# An Accurately Controlled Antagonistic Shape Memory Alloy Actuator with Self-Sensing

**DOI:** 10.3390/s120607682

**Published:** 2012-06-07

**Authors:** Tian-Miao Wang, Zhen-Yun Shi, Da Liu, Chen Ma, Zhen-Hua Zhang

**Affiliations:** Robotic Laboratory, BeiHang University, HaiDian District, 37 XueYuan Road, Beijing 100191, China

**Keywords:** shape memory alloy, self-sensing, hysteresis compensation, instrument

## Abstract

With the progress of miniaturization, shape memory alloy (SMA) actuators exhibit high energy density, self-sensing ability and ease of fabrication, which make them well suited for practical applications. This paper presents a self-sensing controlled actuator drive that was designed using antagonistic pairs of SMA wires. Under a certain pre-strain and duty cycle, the stress between two wires becomes constant. Meanwhile, the strain to resistance curve can minimize the hysteresis gap between the heating and the cooling paths. The curves of both wires are then modeled by fitting polynomials such that the measured resistance can be used directly to determine the difference between the testing values and the target strain. The hysteresis model of strains to duty cycle difference has been used as compensation. Accurate control is demonstrated through step response and sinusoidal tracking. The experimental results show that, under a combination control program, the root-mean-square error can be reduced to 1.093%. The limited bandwidth of the frequency is estimated to be 0.15 Hz. Two sets of instruments with three degrees of freedom are illustrated to show how this type actuator could be potentially implemented.

## 1. Introduction

With the growing demand for miniaturization, like that of medical devices for minimally invasive surgery, many unconventional actuators have been developed, typically possessing the following characteristics: high energy density, specific power and ease of fabrication [1]. For improved compactness, the use of self-sensing is required to maximally reduce the amount of additional sensors, which would also increase the robustness and fail-safety of a given system. Some advanced materials with self-sensing capabilities, including piezoelectrics [2], (IPMC) polymers [3], electromagnetic materials [4] and Shape Memory Alloys (SMAs) [5], have been used as actuators. The measured variables and controlled variables of an actuator must exhibit deterministic and repeatable behavior to attain accurate control.

SMA is a metal that exhibits a crystal transition between a high-temperature austenite phase and a low-temperature martensite phase. At low temperature SMAs exhibit a small Young's modulus and thus can be stretched easily; moreover, they can contract back to their original shape, overcoming roughly twice the pulling force when heated. The maximum recoverable strain is more than 4% of the original length. SMA actuators have been adapted for flexible and miniaturized applications due to their high energy density, mechanical simplicity, compactness and clean operation. Some mini-actuators of miniature mechanical devices have already been used, but without accurate control [6]. Experiments show that SMA actuators can be accurately controlled by position [7] and force feedback [8].

Moreover, when a SMA changes its shape by metallographic transformation, the electrical resistance also undergoes an observable change, which is much more significant than the resistance change due to the alloy's shape. Some research has been performed to determine the relationship between the strain and resistance of SMAs, but the associated mathematical modeling is difficult to perform when the components of the SMA are different [9]. The use of resistance as a sensor has been studied by several authors [5,9–14], and most of these studies focus on wire-spring or wire-constant force pairs. When a system requires opposite pulling units with sufficient stiffness, the size of the spring unit always limits the applied configuration of the SMA actuators. Compared with those actuators, two antagonistic SMA wire actuators or multi-wire actuators with self-sensing capabilities have a clear advantage in terms of miniaturized applications. Because both SMA wires have a nonlinear stress-strain relationship with changes in temperature, there is a need to further study the strain-resistance relationship affected by varied pre-strain and the actual inner-stress between two SMA wires.

In this paper, we present our research on SMA resistance feedback control architectures, with respect to an actuator with an antagonistic pair of SMA wires. A new approach for precision sensor-less SMA servo control is proposed, which consists of two components: the hysteresis paths of both wires are modeled by using polynomial functions and a hysteresis model is used to compensate for the heating duty cycle difference (DCD) of the two wires. The model is based on the “Logistic Curve”, which is typically used to model the hysteresis temperature function of transformations. An antagonistic pair of SMA wires makes the actuator more suitable for miniature applications than wire-spring actuators. Two sets of instruments with three degrees of freedom (DOF) actuated by a pair of SMA wires are illustrated to demonstrate their potential applications.

Beginning in Section 2, the experimental setup of the testing platform is described. In Section 3, after a preliminary discussion of the strain-resistance relationship, the effects of the pre-strain and duty cycle of the PWM signal (heating speed) are investigated. Section 4 presents the modeling of the SMA actuator. Based on self-feedback and a DCD compensator, the control scheme is presented in Section 5, and experimental results are discussed. With respect to applications in minimally invasive surgery, Section 6 further demonstrates two sets of 3-DOF instrument concepts. Finally, Section 7 presents the conclusions drawn from this study.

## 2. Experimental Setup

**Figure 1.**
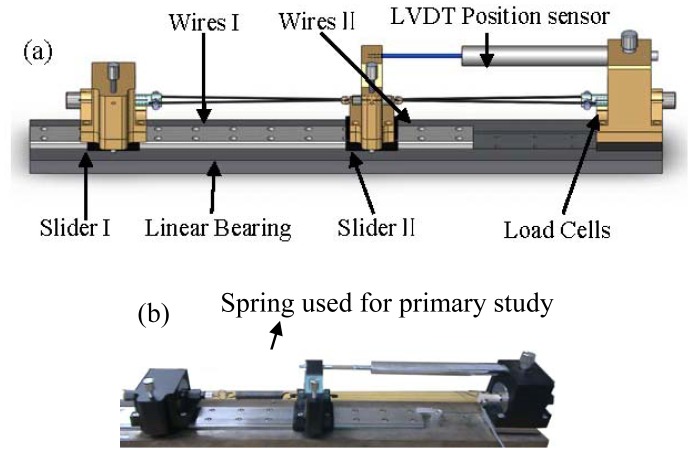
Diagram of experiment setup: the platform.

To study the strain hysteresis curve with respect to both wires' resistance, a testing platform was setup as shown in Figure 1(a): two sliders (slider I with load cell) and another riveted load cell were installed on the linear bearing, and a pair of V-shaped 350 mm long TiNi-based Flexinol^®^-LT SMA wires measuring 0.381 mm in diameter were connected between the slider and load cell by clamps. The V-shaped wire can be approximated as two 175 mm straight wires after ignoring the small angle caused by the screw. A V-shaped wire is more convenient than a straight wire because power can be provided on the same side, and it can be used directly on a 3-DOF instrument. For the two-wires system, slider I will be riveted after two wires have been stretched to a proper length. A linear bearing keeps both sliders moving horizontally. A load cell is attached to one V-shaped wire to denote the contraction force F, with no need to consider the pre-tension force that is always associated with a single-wire system [15]. A linear variable differential transformer (LVDT) position sensor with 10 μm resolution, whose tip is placed against the slider II, is used to measure the displacement of the slider II. In Figure 1(b), one wire has been replaced by a spring for the primary study discussed in Section 3. Notice that the LVDT sensor was used to construct the strain-resistance modulus and validate the control result but not used to feed the signal back to the controller.

**Figure 2.**
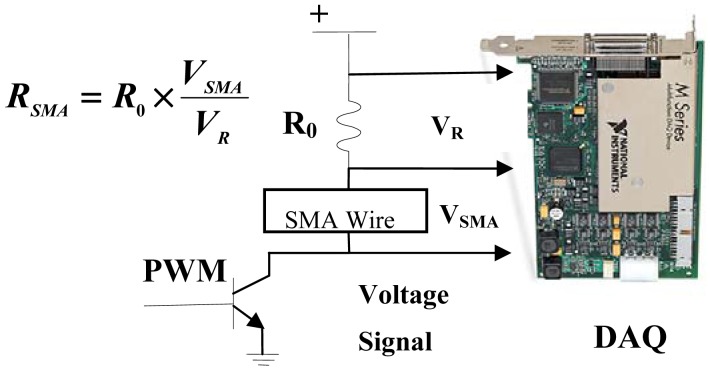
Schematic of the electric circuit.

An electric circuit was constructed as shown in Figure 2: a multifunction data acquisition card (±10 V full-scale range, 18-bit resolution; PCI-6284, NI) is employed to send the PWM signal via the digital output and measure the voltage VR and VSMA via the analog input. A Darlington driver is used as a switching element to control the heating or cooling state of the SMA actuator. An external resistor R_0_ is connected serially to the SMA actuator, which is used to measure the standard resistance. The actual voltage across the SMA wire, VSMA, and the voltage across the external resistor, VR, are measured by the data acquisition card.

All of the resistance values discussed below are the proportional resistance values between the SMA and external resistor.

## 3. Principle of Self-Sensing with Antagonistic Pair Wires

### 3.1. Primary Study on SMA Wire-Spring System

**Figure 3.**
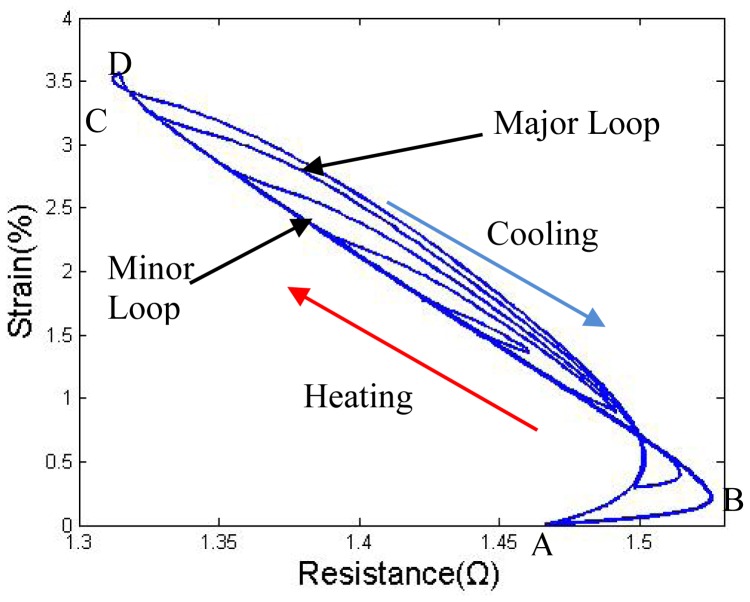
Strain to resistance curve (d = 0.381 mm).

Before beginning the study on antagonistic pair wires, some primary studies on the strain-resistance (S-R) curve of a SMA wire pulled by a spring were carried out. A spring was applied to stretch wire II instead of wire I in the first set of tests. The supply voltage as set to be 7.5 V, and a duty cycle of 25 Hz PWM signal input was varied between 1 and 100%; the pre-tension force was between 18 N and 40 N. As shown in Figure 3, when the pre-tension force is not large enough, the S-R hysteresis curve exhibits a significant hysteresis gap and the difference between the heating and cooling path cannot be ignored during the accurate control of the SMA actuator.

Small duty cycles (80%–30%) results in four minor hysteresis loops. Even when the minor loops are all inside the major loop, a preliminary study shows that it is extremely complicated to describe the minor loops precisely [16].

**Figure 4.**
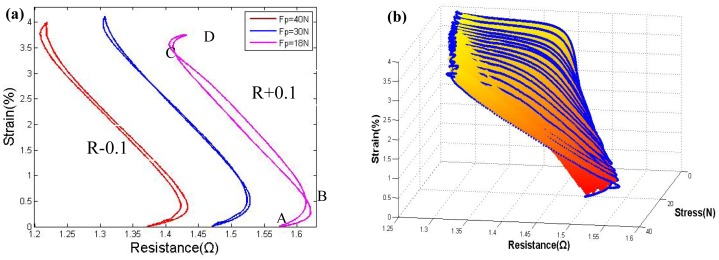
Strain *vs.* resistance curve with different pre-tension forces (d = 0.381 mm): (**a**) classic characteristic shape under different pretension forces; (**b**) 3-D image of strain-resistance-stress relationship.

The results in the study by Lan [10] show that a proper pre-tension force could decrease the gap between the heating and cooling path. We have performed some similar tests. As shown in Figure 4(a), with a 60 N pre-tension force, which indicates a 30 N force on each wire because of the V-shape, the heating and the cooling paths nearly overlap; with a greater pre-tension force, the hysteresis gap start to increase again due to overstress, which also induces an apparent degradation over several cycles. Figure 4(b) shows a 3-D image of the major loop for the strain-resistance-stress relationship; the shape is similar to an open pocket, facing the small-force direction. In both Figures 3 and 4, the slope of the strain-resistance curve of segment BC is the inverse of the slope of segments AB and CD. The resistance increases in AB and CD are due to an initial stage of phase change or overheating without phase change, which normally accounts for 20% of the entire strain. To control this part of the strain curve, some control strategies must applied in the modeling.

After driving the wire by a spring, to minimize the hysteresis gap between the heating and the cooling path, it is necessary to place enough stress on both wires to simplify the control program. Unlike the single-wire system, even though the antagonistic wires are initially taut, both wires will still slacken after being heated and cooled once. It is barely possible to set a pre-tension force on the antagonistic pair wires without an external element. The following subsections discuss the effects of pre-strain and duty cycle on the S-R curve of antagonistic pair wires, which determines the proper pre-strain and actual stress between the two wires. To stabilize the performance of SMA wires, after the testing platform is fixed, all of the data will be obtain after at least 10 training cycles [17].

### 3.2. Effect of Pre-Strain

Flexinol^®^-LT SMA wires could have a strain bandwidth of more than 4%, under the appropriate stress. However, Furuya *et al.* [18] observed that the length of the wire gradually increased during repeated thermal cycling under a given load. Furthermore, Sofla *et al.* [19] have proven that the degradation of a SMA antagonistic actuator depends on the pre-strain. To obtain a wider strain band, we try to investigated the influence of pre-strain and training cycles on the pair of wires.

As mentioned previously, unlike the single wire system, the two-wire system does not feature an initial pre-tension force. Before testing, by pulling slider I, both wires are initially stretched. However, even though the wires are taut at the beginning, after submitting both wires to one heating cycle (though not at the same time), the wires will still slacken after both wires become cool. This slackening of SMA wires is called the two-way shape memory effect. This effect is explained by Kohl [20], who states that that once this property is expressed, the wires are actively lengthened when cooled, even without a tension force. With a proper pre-strain, the slack can be controlled to a small extent.

To make both wires achieve roughly 4% displacement after stretching, the pre-strain must be greater than 4% of one wire's original length. Meanwhile, as mentioned by Ma *et al.* [5], SMA wires always require a few training cycles to exhibit reproducible behavior. To attain a proper working distance and steady state, degradation testing was performed with pre-strains ranging between 4%–5.5%. The drive wire was heated by a 9 V supply voltage and a 25 Hz PWM signal with an 85% duty ratio input while the passive wire was cooled, and roughly 500 training cycles were performed under different pre-strains. The experimental results show that, with 4.6–5% pre-strain, after more than 30 training cycles, the contraction strain will be stable at roughly 3.8% of one wire's original length, and part of the SMA stroke will be compromised to tighten the wire before pulling the slider II. A greater pre-strain will lead to overstress at the beginning of the strain, which sometimes terminates the phase transformation completely, which in turn, leads to distinct degradation, so again more training cycles are required to achieve reproducibility. At a lower pre-strain, it is difficult to maintain a stable displacement of two wires. Without overstress, the contraction strain shows no sign of degradation after sufficient training. After one wire is heated, the two wires become taut, and they will not slacken again without cooling down both wires.

A series of experiments was also performed to determine the pre-strain effect on the relationship between the S-R curves of both wires. An excessive pre-strain makes the gap between the heating and the cooling path wider due to the overstress, and a deficient pre-strain will also make the gap wider because the stress is not large enough.

### 3.3. Effect of Duty Cycle on Strain-Resistance Hysteresis Curves

Same as in the single-wire system, the duty cycle controls the heating speed and the highest temperature SMA wires can withstand. The difference is that the contraction force of the antagonistic pair wires system is not a constant value or changes with a constant Young's modulus, which is due to the non-linear decreased in Young's modulus when materials are cooled. It was concluded from the pre-study [21], that, the phase transition can be caused by both temperature and applied force. To make accurate self-sensed accurate control possible, reproducible strain-resistance curves with a convenient hysteresis gap are a prerequisite.

**Figure 5.**
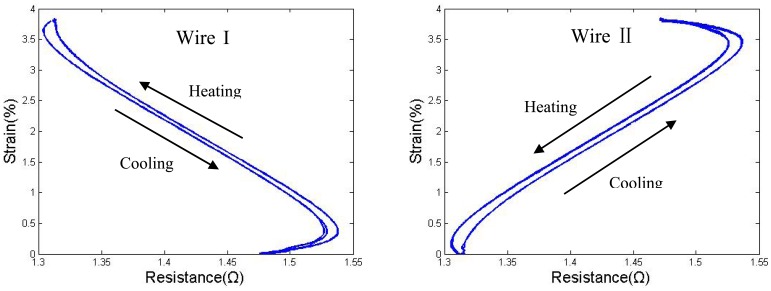
Strain *vs.* resistance curve of wire I and wire II overstressed by a high duty cycle (d = 0.381 mm).

Some investigations have been performed to this end. The supply voltage was set to 9 V; this is higher than the voltage for wires under a constant force, in case the wires require a higher temperature to surmount the large pulling force. The pre-strain was set to 4.8% in this group of tests, and the experiments were carried out after 30 training cycles. The driving procedure of the first group was as follows: when one drive wire is heated by a roughly 99% duty cycle, the passive wire is heated by a roughly 1% duty cycle, which is performed simply to monitor the resistance; in the return cycle, the duty cycle is exchanged. The S-R curves of both wires after three cycles (after training and both cooling down once), are shown in Figure 5: the zero points of strain determine the contraction limitation position of wire II. All of the wires used in the study were submitted to 30 training cycles before testing.

As shown, the S-R curves of both wires became stable and repeatable, even though the hysteresis gap between the heating and the cooling paths, which was induced by the overstress, was too wide to be ignored. Wire I and wire II show nearly the same properties in their strain-resistance curves; only the directions are reversed. Thus, there is no need to show both wires' S-R curves in every analysis in cases in which they are similar, and most of the discussions below will simply show the results of wire I.

**Figure 6.**
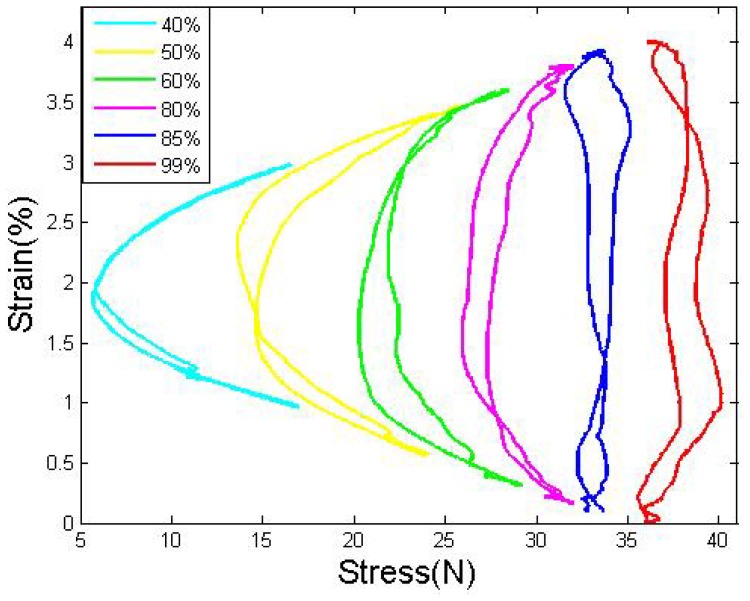
Strain-stress curve under different drive duty cycles.

The duty cycle, which determines the equivalent current of the drive wire, directly controls the actual stress applied to the wires. A higher duty cycles leads to a faster heating speed and larger contraction strain, which could increase the antagonistic stress. To avoid overstress and to attain the best frequency, we tried to determine the most appropriate duty cycle. More tests, were conducted to determine, the influence of the duty cycle on the system: the duty cycle of the drive wire varies between 40% and 99%, and the passive wire is kept heated by a 1% duty ratio. The flexure load in Figure 6 shows a regular distribution, and clearly increases with duty cycle. Before the duty cycle exceeds 85%, the stress in the middle position is smaller than the stress in the edge position because the cooling is quicker than the heating; after the duty cycle exceeds 85%, the stress at the edge position becomes equal to or larger than at the middle position because heating is equal to or quicker than the cooling. The same stress was applied to both wires.

**Figure 7.**
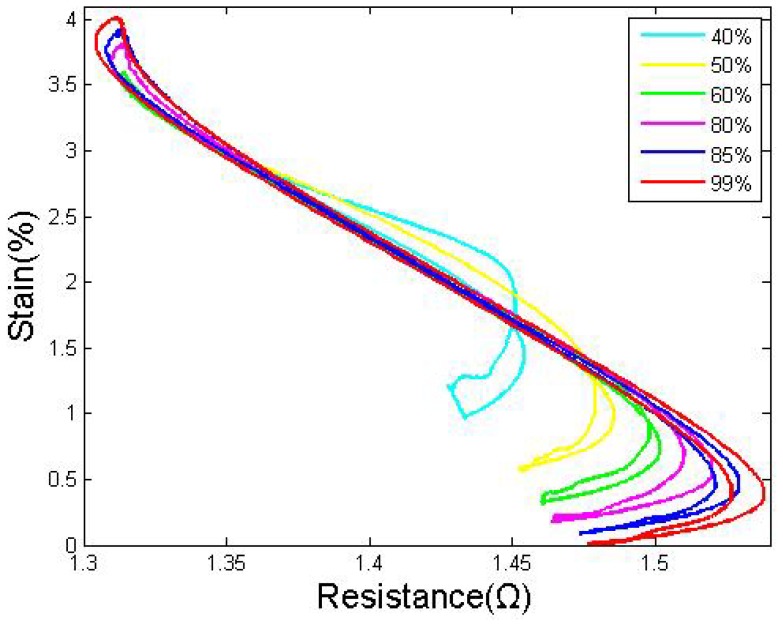
Strain to resistance curve under different drive duty cycles (d = 0.381 mm).

By studying the S-R curves of SMA wires with different drive duty cycles, Figure 7 shows that: before the duty cycle exceeds 85%, the strain grows significantly with the duty cycle; meanwhile, the hysteresis gap between the heating and the cooling path decreases. When the duty cycle is equal to 85%, the gap between the heating and cooling paths is minimized. After the duty cycle exceeds 85%, the strain limitation increases slightly, but the hysteresis gap starts to increase again due to the overstress.

It is believed that the overstress might be caused by the slow cooling speed of the passive wire. A higher duty cycle indicates a higher heating speed, but the cooling speed of the passive wire does not change, which forces the drive wire to get overcome the higher stress to contract before the passive wire completes its phase transformation. The overstress affects not only the S-R curve but also causes the degradation of the SMA wires, which should be avoided.

As shown in Figures 6 and 7, in a certain range, the stress could be considered roughly constant, and normally in this range the slider has compare large strain bound also with small hysteresis gap. Under this voltage, we conclude the most convenient duty cycle is between 82%–87% under this voltage.

## 4. Modeling of SMA Actuator

### 4.1. Modeling of Duty Cycle Difference Hysteresis

**Figure 8.**
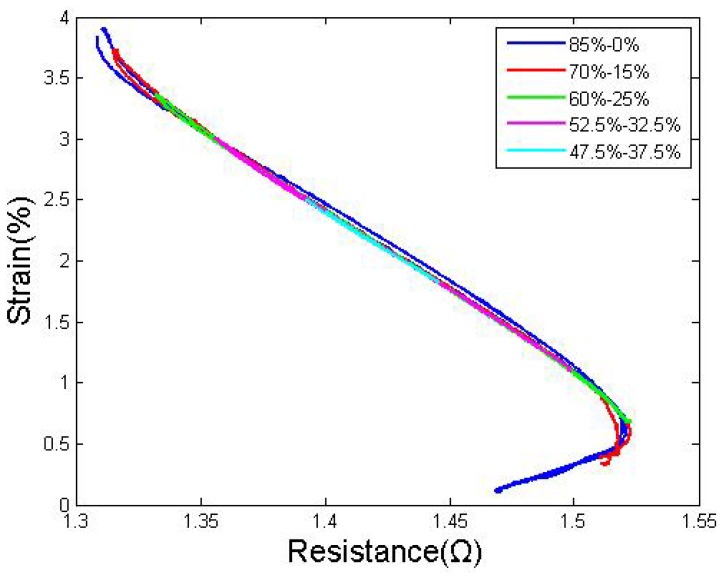
Same summation of duty cycles under different DCD.

Further research has been performed to characterize the system in which both wires are heated at the same time with different duty cycles. After several series of tests, we discovered that if the sum of both wires' duty cycles is constant and greater than 82%, the DCD of the two wires will not affect the stress or the S-R curves. The DCD could simple change the strain value. As shown in Figure 8, to arrive at a reasonable conclusion, the sum of the duty cycles of both wires was maintained at 85%, and the duty cycle of the drive wire was varied between 85% and 47.5%.

**Figure 9.**
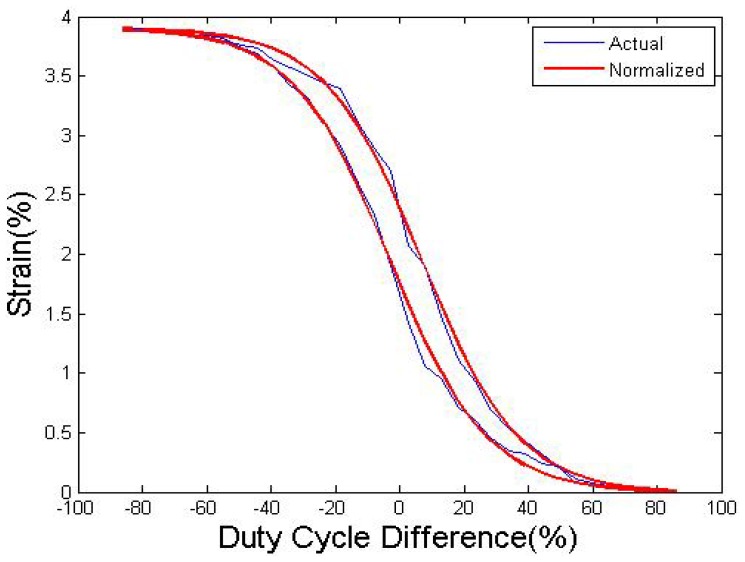
Strain to DCD curve (d = 0.381 mm).

To further determine the relationship between the strain and DCD, a continuous strain-DCD curve was established, as shown in Figure 9. The duty cycle changes at a very slow speed to make sure the phase transformation is completely. The horizontal axis is the DCD of wire I and wire II, which varies from −85% to 85%. The strain-DCD curve shows typical hysteresis characteristics, which makes it difficult to use duty cycling to control the movement directly. In addition, the contraction speed could become quite low due to the inverse force from the antagonistic side when the DCD is too small.

The integral of the curve is similar to the classical ratio-temperature transformation, and the strain-DCD relationship could be described by the same function. In this case, the “Logistic curve” is used to simulate the strain change with DCD. The heating curves and the cooling curves are modeled respectively by equations (1) and (2), respectively. The duty cycles of wire I and wire II are modeled by equations (3) and (4):

(1) $$dD_{h}=3.91/(1+\exp(0.066*(S+3)))-0.01$$

(2) $$dD_{c}=3.92/(1+\exp(0.066*(S-7)))-0.015$$

(3) $$D_{I}=(dD+85)/2$$

(4) $$D_{\mathrm{II}}=(85-dD)/2$$

where *dD* is the DCD of wire I and wire II, *dD_h_* and *dD_c_* are the *dD* values of the heating and cooling curve, and *D* are the duty cycles of the wires. The mathematical models (1)–(4) only describe the major hysteresis loop, but as mentioned in last paragraph, when the DCD is too small, the contraction speed also becomes quite low, and it may be more appropriate to use the major loop to improve the accuracy and simultaneously preserve a convenient speed.

### 4.2. Modeling of Self-Sensing Properties

**Figure 10.**
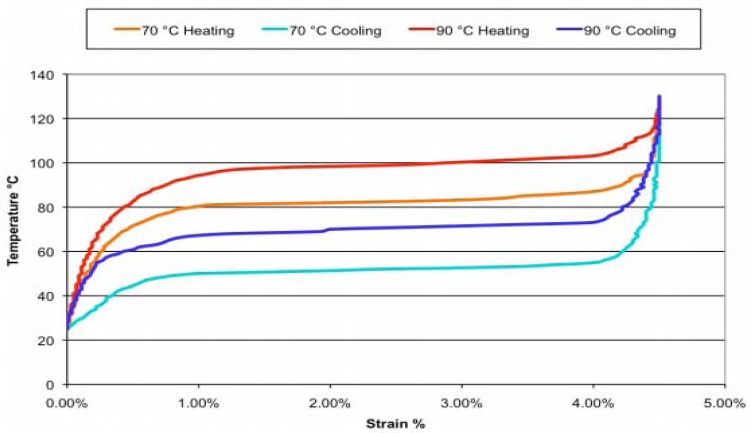
Relationship between strain and temperature for Flexinol^®^.

Some studies on materials properties [22] proved that, the strain-temperature curve of SMA wires exhibits hysteresis. Figure 10 shows the relationship between the temperature and strain of the LT and HT of Flexinol^®^ wire, as published in the product specifications [23]. For the heating process, the strain is small until a temperature above 70 °C is reached; for the cooling process, the strain is large until reaching a temperature below 50 °C. During the experiment, we discovered that the time required to cool down the last 0.2% of the strain was nearly the same as that required to cool down the remaining strain. To obtain a faster response, we considered keeping the passive wire just below the transition temperature instead of completely cooling it down, which may reduce the time needed to cool the passive wire and heat the drive wire.

**Figure 11.**
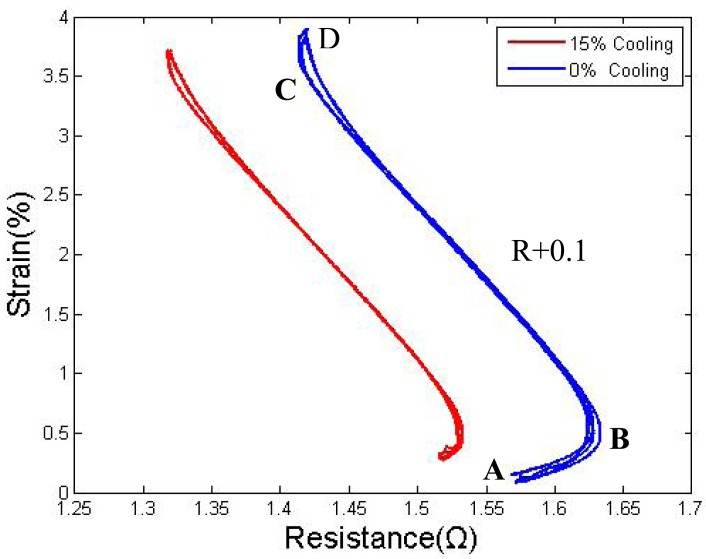
Strain to resistance curve: 15% duty cycle cooling and 0% duty cycle cooling.

Some tests have shown that a duty cycle of approximately 15% it is enough to keep the wire temperature just below the transformation point, which is approximately 40 °C–45 °C. Considering the results of Section 4.1, the actuator is controlled as follows: the drive wire is heated using an 85% duty cycle at the beginning, and the stretched wire is cooled down; after the passive wire is cooled to the properly resistance, it will be heated using a low duty cycle of 15% to maintain the temperature. To maintain this position and prevent the formation of overstress, the active wire will also be heated at a relatively low duty cycle of 70%. The results for this cycling are shown in Figure 11—when the passive wire is kept warm, the AB and CD segments become narrow because the active wire is not heated to its limitation position to avoid overstress, and the displacement is approximately 0.4% of the original length less. The strain distance becomes slightly shorter, but the time required to complete one cycle is reduced by nearly 50%.

**Figure 12.**
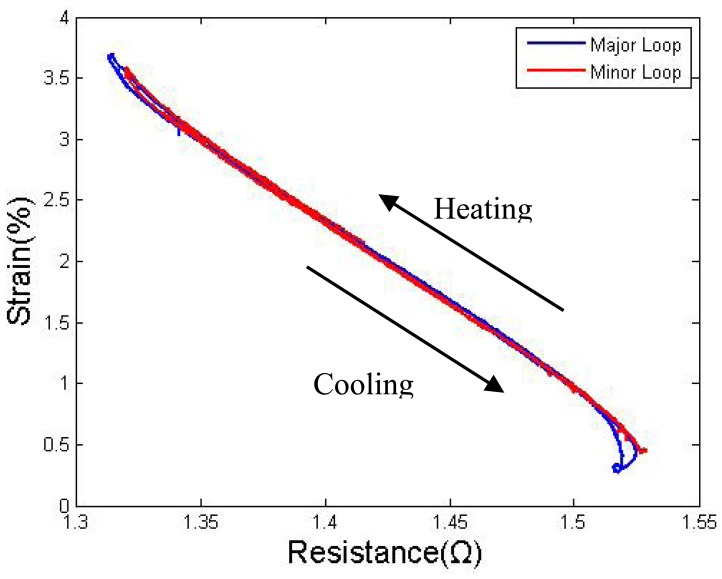
Strain to resistance curve: major and minor loop.

To convert the electric resistance to strain in the self-sensing control system, normally an S-R hysteresis model needs to be established to provide the strain information by sensing the SMA resistance. Some advanced hysteresis models, which can model the major and minor loops at the same time, can be widely found in the literature [12]. However, as Lan reported [10], with a proper pre-tension force, the heating and cooling paths are so close to each other that it makes the modeling of the minor loop unnecessary. The results shown in Figure 12 also support this conclusion. A pair duty cycle of 85%–0% was used to actuate to a target, switching the duty cycle of the two wires upon semi-cycle completion. Eight targets resulted in three minor hysteresis loops, and all three minor loops were covered by the major loop, showing only a few differences occurred at the two ends.

In the antagonistic pair wires drive system, the S-R curves of the two wires are corresponding, and the AB segment of wire I corresponds to the CD segment of wire II. For the case in which the two wires could both feedback the resistance value, to simplify the control model and avoid the transition segment, segment BD for the heating path and segment DC for the cooling path of both wires were used to construct the entire control model.

Considering these factors, a polynomial model with one-to-one mapping is sufficient to describe the path of the major loop. The MATLAB^®^ “polyfit” function was used to obtain the coefficients of the polynomials. Seventh-order polynomials were verified by testing to have sufficient accuracy. The approximate functions for the heating and the cooling paths of both wire I and wire II are denoted as

(5) $${\text{R}}_{\text{Ih}}={\text{r}}_{\text{Ih}}(\text{S});{\text{R}}_{\text{Ic}}={\text{r}}_{\text{Ic}}(\text{S});{\text{R}}_{\text{IIh}}={\text{r}}_{\text{IIh}}(\text{S});{\text{R}}_{\text{IIc}}=r_{\text{IIc}}(\text{S})$$

where R is the resistance of SMA wires, h and c indicated heating and cooling curve, respectively, and S is strain. As mentioned in section 2, a pair of V-shaped 350 mm long TiNi-based Flexinol^®^-LT SMA wires measuring 0.381 mm in diameter were considered. Figure 12 shows the pair's actual S-R curve at 4.8% pre-strain. The seventh-order polynomial fitting functions were:

(6) $${\text{R}}_{\text{Ih}}={\text{r}}_{\text{Ih}}(S)=0.426S^{7}-7.190S^{6}+51.865S^{5}-207.201S^{4}+495.133S^{3}-707.866S^{2}+560.613S-188.168$$

(7) $$R_{\mathrm{Ic}}=r_{\mathrm{Ic}}(S)=0.403S^{7}-7.035S^{6}+52.428S^{5}-216.116S^{4}+532.102S^{3}-782.491S^{2}+636.255S-219.052$$

(8) $${\text{R}}_{\text{IIh}}={\text{r}}_{\text{IIh}}(\text{S})=-0.372S^{7}+5.868S^{6}-39.426S^{5}+146.379S^{4}-324.479S^{3}+429.692S^{2}-314.856S+99.803$$

(9) $${\text{R}}_{\text{IIc}}={\text{r}}_{\text{IIc}}(\text{S})=0.262S^{7}-4.375S^{6}+30.862S^{5}-119.101S^{4}-271.457S^{3}-365.104S^{2}+268.137S-81.598$$

**Figure 13.**
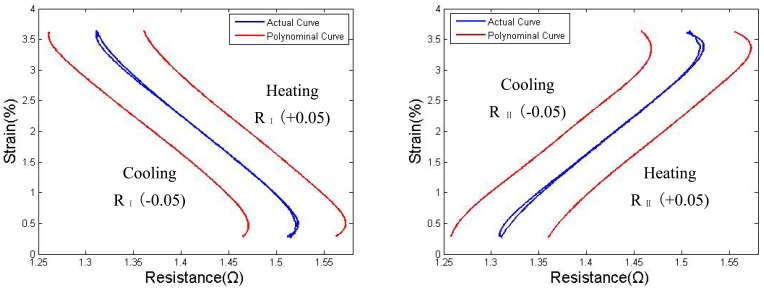
Actual and polynomials S-R curve: wire I and wire II.

As shown in Figure 13, the functions r_h_(S) and r_c_(S) were shifted by R = +0.05 and R = −0.05 and then plotted. In this case, once a strain S_t_ has been targeted, the corresponding resistance R_S_ will be compared with the self-sensed resistance to modify the output signal.

In the control scheme, the fitting functions are used according to the scheme presented in Table 1.

## 5. Controlling with Self-Sensing Feedback and Hysteresis Compensation

### 5.1. Method

By using the polynomial model to obtain the feedback signal, a PID controller was also combined to further control the strain. After using the polynomial model of Equations (6–9) to obtain the feedback signal, a PID controller was used to generate the duty cycle D1 of the PWM signal to implement the tracking. The expression of the appropriate duty cycle is expressed as follows:

(10) $$D(n)=k_{p}e(n)+k_{i}\{T_{i}[e(n)-e(n-1)]+S(n-1)\}+k_{d}(e(n)-e(n-1))$$

where K_p_ is the proportional parameter; K_i_ is the integral parameter; K_d_ is the differential parameter; the error e(n) is the difference between R_S_ and R_i_; T_i_ is the sampling time. The parameters K_p_, K_i_ and K_d_ of the PID controller are auto-tuned by fuzzy logic rules. We adopted the fuzzy reasoning method to determine the parameters, which are based on the values of e(n) and e(n − 1). The parameters are tuned between K_p_ = 0–5, K_i_ = 0–0.9 and K_d_ = 0–0.2. Because the wires' conditions are symmetrical, they use the same PID parameters. It should be noted that, under these parameters, the duty cycle of the passive wire is kept at roughly 1% until the resistance reaches the target value or the value of point B; then it is increased to 15%.

**Figure 14.**
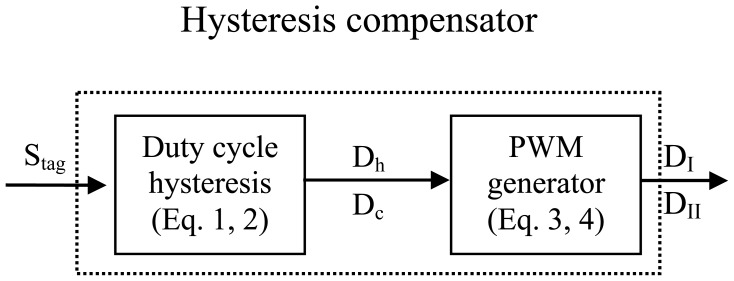
Hysteresis compensation diagram.

**Figure 15.**
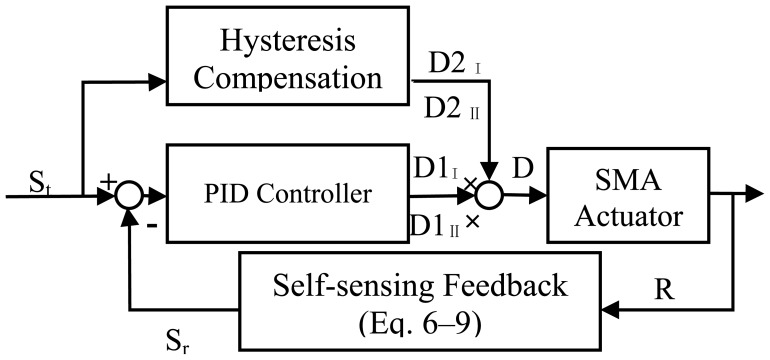
Flow Diagram of SMA actuator control.

Because SMA actuators are a highly nonlinear system, even by using PID control combined with resistance feedback, the results show that simulaneously avoiding overheating and achieving higher speeds at same time is still difficult. A duty cycle hysteresis compensator was designed to compensate the hysteresis, which could nearly eliminate overheating. The compensation scheme for controlling the SMA actuator displacement is depicted in Figure 14. The input of the compensator is the target strain Stage, S_tag_, two blocks that calculate the corresponding duty cycle of the input PWM signal for both wires, D_I_ and D_II_. The feed-forward compensator estimates the duty cycle required to heat and cool the pair of antagonistic wires, which makes slider II move to the target position. The corresponding DCD was obtained by the polynomial model described by Equations (1,2). Equations (3,4) was used to calculate the duty cycle D2 of both wires' input PWM signal. As shown in the flow diagram in Figure 15, the SMA actuator is controlled by the PWM signal with duty cycle D, which is computed by multiplying D1 and D2. The parameters of the PID controller were varied between K_p_ = 0–5.8, K_i_ = 0–1.2 and K_d_ = 0–0.11.

### 5.2. Experimental Results

**Figure 16.**
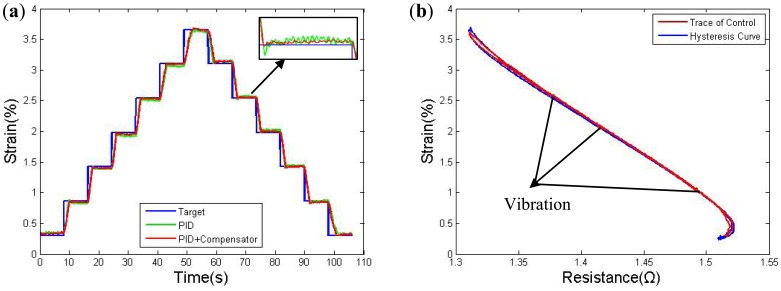
(**a**) Multi-steps response; (**b**) S-R Trace of multi-steps' PID + compensator control response.

Two tests were performed to determine the accuracy of the proposed control scheme. The first experiment used a multi-step control signal. Figure 16(a) presents the measurement results for the LVDT, which shows the target signal and the control result obtained using only resistance feedback PID control and simultaneously using both the PID controller and the compensator. The PID controller parameters were not constant in the two groups. The responses were tested after three cycles until the stress becomes stable. The increment in each step is 0.55% strain over roughly 8 s; the target stain and actual strain are very close to each other over most of the effective distance, but the error increases from an average value of 0.02% strain to 0.04% strain nearby the edge of the bandwidth. As indicated by the result, the tracking error is clearly large for the PID controller due to overheating, and the track also exhibits a larger wave in each step. Figure 16(b) further shows the trace points of the strain to resistance relationship during the multi-step control experiment. The points are recorded every 0.04 s. The results show that, the strain value changes with vibration when it stops at each step, which is caused by slight overheating. However, the vibration is weak, which might not affect the accuracy much in case the swings are less then ±0.06%.

To study the difference between the target strain and the actual strain in the different control models, we use the root-mean-square error (RMSE) of the multi-steps response to evaluate the accuracy of the models:

(11) $$\text{RMSE}=\sqrt{\sum_{i=1}^{n}\left({\left(\left|S_{\text{tag}}-S_{i}\right|/S_{\text{tag}}\right)}^{2}/\left(n-1\right)\right)}\times 100\%$$

where S_tag_ is the target strain of each steps, S_i_ is the actual strain tested by LVDT after the actual resistance equal to the target resistance, which is obtained from Equations (6–9), until the end of each step; n took the value of 13 which is the step number in this test. The RMS tracking errors for the single-wire feedback model, the two-wire feedback model and the proposed model were 2.745%, 1.628%, 1.093%, as shown in Table 2. The smaller RMS error confirms the effectiveness of the proposed model.

**Figure 17.**
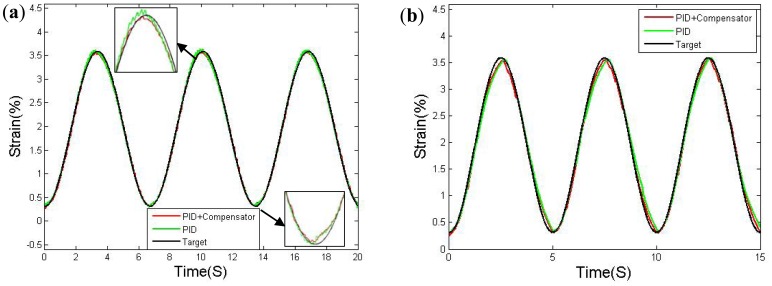
Sinusoidal tracking response: (**a**) 0.15 Hz; (**b**) 0.2 Hz.

The second test featured a sinusoidal control signal, the testing results measured by the LVDT sensor after three cycles of training (after 30 cycles of primary training) are depicted in Figure 17. The target value can be treat as the resistance translate value. Tests were performed using 0.15 Hz and 0.2 Hz sinusoidal waves. As shown, the tracking errors improved when the feed-forward compensator was incorporated, even the speed was slightly reduced at the peaks. The 0.2 Hz wave shows a visible error of 0.16% nearby the peaks, much higher than the 0.06% error in the 0.15 Hz wave. The results indicate that the 0.15 Hz is the limited bandwidth of this SMA actuator.

**Figure 18.**
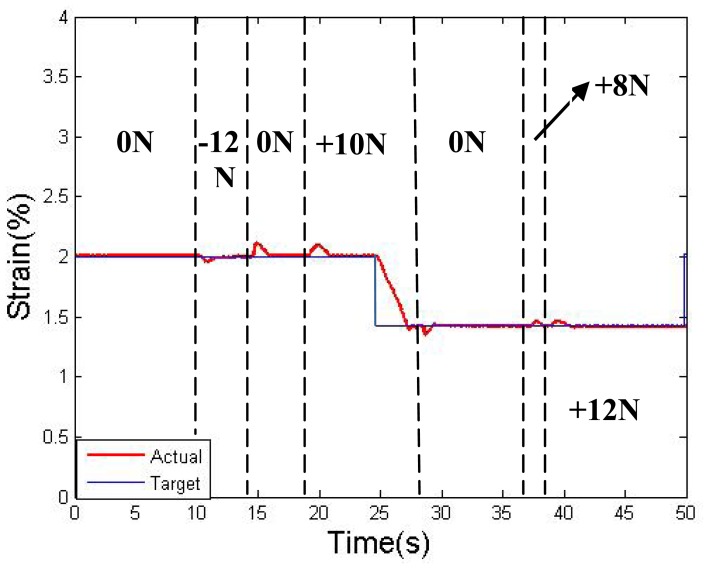
Multi-step response under force disturbance.

Further disturbance tests were performed during the step-response experiments; the results are shown in Figure 18 for the LVDT. Extra force has been applied to the slider, from +12 N to −12 N. The force direction is parallel to the linear bearing, where + represents the direction from wire II to wire I, and − represents the opposite direction. The results show that, with a disturbance under 12 N, the slider shows roughly 0.15% strain wave, but the wave is eliminated in 1.5 S by the rectification of the resistance feedback. After the disturbance is removed, a similar jump in the strain will also occur in the opposite direction. The experiment result proves that, the control result could still be quite accurate even with 12 N of extra stress. In this case, we can assume that when the pre-strain (pre-stress) is large enough, the two-wires actuator can operate under a load of less than 12 N. It should be noted that, the ability to resist interference depends on the wire diameter, which is limited by the available stress range. The diameter of the SMA wires should be chose according to the output requirement of the actuator, and the control model does not change much with diameter.

## 6. Application to a Three DOF Instrument

**Figure 19.**
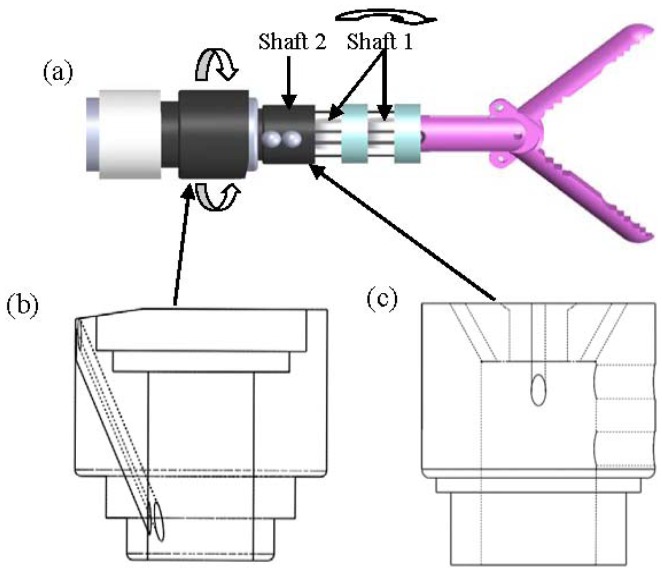
Concept definition of two shafts: assembly of vertebrae and wires.

Based on the results presented in the preceding section, this section further demonstrates two mechanisms designed for an antagonistic SMA actuator. Both mechanisms have three DOF, including two DOF for the pitch and yaw of the wrist and one DOF for grasp. The design is planned to be used as a medical robot instrument in the future. Figure 19(a) depicts a traditional design including a coupling of two rotation shafts: shaft 1 offers a rotation DOF for the yaw of the wrist, for which a commercially available plastic bearing is planned to be used; shaft 2 is composed of two ceramic columns and three discs, whose total pitch is approximately 90°. Each disc in shaft 2 has eight holes for the SMA wires to go through. To avoid interference between the drive wires of the two shafts, some slots were made to conduct the drive wires near the axis of the tube (Figure 19(b,c)). Two extra wires were used to control the opening and closing of the grip.

Unlike the two shaft design, to orientate a system in all directions in a compact volume, a ball-joint link may be another interesting solution. van Meer *et al.* [24] proposed a multi-channel plastic joint with a ball-joint link, in which three drive wires were connected on distal motors to command the angulations of the joint. This device shows good volume expenditure and sufficient output as well.

**Figure 20.**
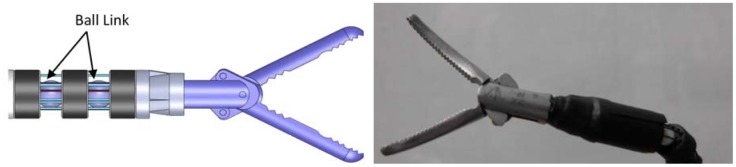
Concept definition of ball-joint: assembly of vertebrae and wires.

In our design, the distal motors were removed, and the driving force was directly derived from the contraction of the SMA wires, which make the whole instrument more compact. Also the number of drive wires is set to be four to produce two antagonistic pairs of wires. As shown in Figure 20, six SMA wires (four for ball-link, two for grip) are set to supply the driving force, and another four stiffness wires are set between the drive wires to offer enough axial and lateral stiffness. To maintain the pre-strain, a screw unit was adding to the end of wires, which was used to pull SMA wires to a proper length.

In both designs, the wires are connected to the top disc, but usually the snake-like joints can assume an arbitrary position without properly pulling. After the concepts have been fully developed, experiments will be conducted to determine the need for using two or four more wires connected to the middle disc. In theory, both designs should operate without any interference between the two groups of drive wires, though interference could still occur due to inaccurate wire placement. Some compensation to eliminate this interference will be added to the control model in the future.

## 7. Conclusions

This study realized the accurate control of an actuator consisting of an antagonistic pair of SMA wires by self-sensing feedback with a hysteresis compensator. The effect of pre-strain was first investigated to make sure that the proper deformation of both wires could be maintained after degradation. Under different duty cycles, both the strain-resistance and strain-stress curves were compared to determine the duty cycle required to simultaneously obtain high speed and avoid overstressing. Under a voltage of 9 V, an 85% duty cycle is suggested to obtain nearly no strain gap in the middle part of the curve. The strains-resistance curves were further shown to be independent of changes in the DCD, when the sum of the duty cycle of both wires was held constant at 85%. Based on this property, a forward feedback compensation of strain to DCD hysteresis model was proposed. Because the strain gap of the major loop is manipulated to be small enough, the self-sensing model was constructed by fitting the major loop to a polynomial function. To expand the control available deformation range as much as possible, a control strategy based on four fitting functions of two wires was applied. Step-response control experiments showed that the root-mean-square error of the proposed method is less than 1.1%, and the external interference can be efficiently eliminated at loads below 12 N. Sinusoidal tracking experiments showed that a 0.15 Hz wave could be considered as the frequency bandwidth of this actuator. Two 3-DOF SMA actuated mechanisms were illustrated to demonstrate the accurate controlled over the antagonistic wires for potential practical applications.

Relative to previous studies [9–14], the major contribution of this work was the application and control of antagonistic pair wires by resistance feedback. By properly pre-straining the system, a control model was developed using simpler methods than those used in a previous study [5].

To apply the actuator in practice, further mechanism analysis must be performed in the future. As discussed in [14], the application of a proper torque applied onto the wires might guarantee more accurate resistance feedback. Moreover, the current control scheme may be affected by the ambient changes in temperature or thermal conductivity. Future research directions should include compensation of the proposed control scheme against such changes.

## Figures and Tables

**Table 1.** Fitting functions correspond with control scheme.

**Resistance Range & Drive wire**	**DB & I**	**AB & I**	**DB & II**	**AB & II**
Function	(6)	(7)	(8)	(9)

**Table 2.** RMS errors of different control models.

**RMSE**	**Single wire Feedback control**	**Two wires Feedback control**	**Proposed control**
%	2.745	1.628	1.093
